# Supplementary material for: Carbocyanine-Based Optical Sensor Array for the Discrimination of Proteins and Rennet Samples Using Hypochlorite Oxidation
Source: Sensors (Basel). 2023 Apr 26;23(9):4299. doi: 10.3390/s23094299 (PMC10181777; doi:10.3390/s23094299)
Supplement: Supplementary file 1 [file sensors-23-04299-s001.zip › sensors-2327755-supplementary.pdf]

Supplementary Materials to paper

Carbocyanine-based Optical Sensor Array for the Discrimination of Proteins and Rennet Enzymes Using Hypochlorite Oxidation

by Anna V. Shik, Irina A. Stepanova, Irina A. Doroshenko, Tatyana A. Podrugina, and Mikhail K. Beklemishev*

[1] *Department of Chemistry, M.V.Lomonosov Moscow State University, GSP-1, Leninskie Gory, 1–3, 119991 Moscow, Russia*

Spectral data for IR-783 dye

4-((*E*)-2-((*E*)-2-(2-chloro-3-((*E*)-2-(3,3-dimethyl-1-(4-sulfonatobutyl)-3*H*-indole-1-ium-2-yl)vinyl)cyclohex-2-en-1-ylidene)ethylidene)-3,3-dimethylindolin-1-yl)butan-1-sulfonate sodium

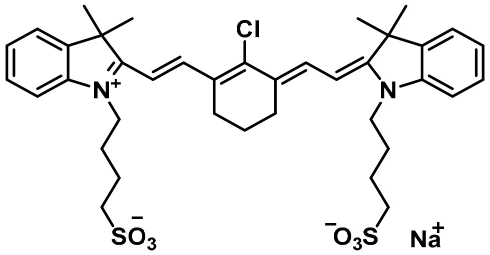

IR-783 was prepared according to paper: Okoh, O.A.; Bisby, R.H.; Lawrence, C.L.; Rolph, C.E.; Smith, R.B. Promising near-infrared non-targeted probes: benzothiazole heptamethine cyanine dyes. *J. Sulfur Chem*. **2014**, *35*, 42–56. https://doi.org/10.1080/17415993.2013.778258.

Yield was 0.43 g (49%), dark green powder. λ_{abs} = 780 nm (in methanol). λ_{fl} = 799 nm (in ethanol).

1H NMR (400 MHz, CD_3OD, δ, ppm, J/Hz): 1.73 (s, 12 H, 2 $C(CH_3)_2$), 1.90 - 2.04 (m, 10 H, 5 CH_2), 2.73 - 2.79 (m, 4 H, 2 CH_2), 2.89 (t, 4 H, $^3J_{HH}$ = 7.12, 2$CH_2SO_3^-$), 4.22 (t, 4 H, $^3J_{HH}$ = 7.12, 2 CH_2N^+), 6.33 (d, 2 H, $^3J_{HH}$ = 14.06, 2 =CH), 7.27 (t, 2 H, $^3J_{HH}$ = 7.34, Ar), 7.34 - 7.45 (m, 4 H, Ar), 7.51 (d, 2 H, $^3J_{HH}$ = 7.34, Ar), 8.44 (d, 2 H, $^3J_{HH}$ = 14.00, =CH).

13C NMR (100 MHz, CD_3OD δ, ppm): 22.28, 22.72, 23.75, 27.45 (all CH_2), 28.50 ($C(\underline{C}H_3)_2$), 45.20 ($\underline{C}(CH_3)_2$), 50.77 (CH_2N^+), 51.92 ($CH_2SO_3^-$), 102.64 (CH), 112.54 (Ar), 123.61 (CH), 126.63, 128.43, 130.07 (all Ar), 142.74 ($\underline{C}$=C(Cl)-$\underline{C}$), 143.75, 145.72 (all Ar), 151.22 (C-Cl) 174.32 (C=N).

IR, ν/cm^{-1}: 1029.32 (C-Cl), 1166.24 (S=O), 1130.01, 1245.31 ($CH_2SO_3^-$), 1548.56 (C=N).

Table S1. An example of a data table used in the discrimination of model proteins. The intensities in the table were obtained using ImageJ software for the images captured at different reaction times. Only the upper left corner of the table is shown. R, G, B refer to the red, green and blue channels of visible light images

	A	B	C	D	E	F	G	H	I	J	K
1		IR-783, visible									
2	Sample	1 min R	1 min G	1 min B	3 min R	3 min G	3 min B	5 min R	5 min G	5 min B	9 min R
3	Blank	179,329	164,896	94,29	182,265	170,799	102,757	182,5	172,156	109,961	178,25
4	Blank	175,833	160,145	88,946	179,066	166,407	97,831	179,352	168,328	105,727	175,164
5	Blank	171,951	157,776	89,035	175,017	163,319	98,005	176,242	166,438	107,18	172,152
6	Blank	169,255	154,538	84,32	172,144	159,854	93,554	173,758	163,902	102,789	170,16
7	Blank	164,419	148,954	80,544	168,675	156,039	89,039	170,113	158,895	95,391	166,25
8	Blank	163,183	147,389	76,389	165,866	153,045	85,041	167,668	155,68	90,941	163,645
9	BSA	154,015	147,645	95,37	177,298	160,984	90,057	180,816	163,469	91,055	178,055
10	BSA	143,404	137,779	92,464	170,217	153,498	83,077	177,152	159,305	86,031	175,277
11	BSA	145,506	136,597	89,199	169,314	152,079	81,124	174,086	156,176	83,375	170,77
12	BSA	123,73	125,559	93,968	152,941	141,834	83,926	165,543	149,605	80,426	166,961
13	BSA	121,258	126,005	93,752	150,711	142,825	84,59	161,34	147,645	78,301	164,434
14	BSA	125,926	124,645	85,42	152,924	139,095	74,275	162,34	145,297	74,91	160,918
15	HSA	136,009	123,964	95,227	169,118	145,894	84,164	180,672	160,156	87,27	178,613
16	HSA	129,796	119,841	97,232	163,347	139,546	88,912	175,609	153,953	85,844	176,625
17	HSA	132,723	121,095	94,33	165,057	142,883	86,771	174,578	154,801	80,719	172,906
18	HSA	122,438	112,918	90,913	156,731	132,483	80,942	168,336	147,523	79,371	168,789
19	HSA	123,863	113,425	92,207	156,244	132,248	83,715	166,375	145,078	78,688	166,254
20	HSA	113,826	108,06	89,639	146,256	122,474	81,215	157,613	134,668	79,051	161,688
21	Lysozyme	124,867	129,869	96,666	157,897	143,081	82,407	172,574	154,574	79,707	174,211
22	Lysozyme	124,691	133,236	97,808	152,157	144,889	86,603	165,402	151,918	81,375	169,203
23	Lysozyme	127,111	130,231	96,118	159,374	143,73	84,234	169,594	151,438	75,746	170,75
24	Lysozyme	117,079	124,369	91,718	146,548	134,493	77,98	160,07	143,516	74,996	163,68
25	Lysozyme	108,473	121,907	91,589	138,581	131,397	78,513	152,363	139,281	75,383	158,094
26	Lysozyme	115,493	124,393	88,407	143,858	135,612	75,651	155,508	141,555	70,668	159,375
27	Globulin	124,131	127,866	102,361	166,602	147,57	86,847	178,012	158,828	84,918	177,918

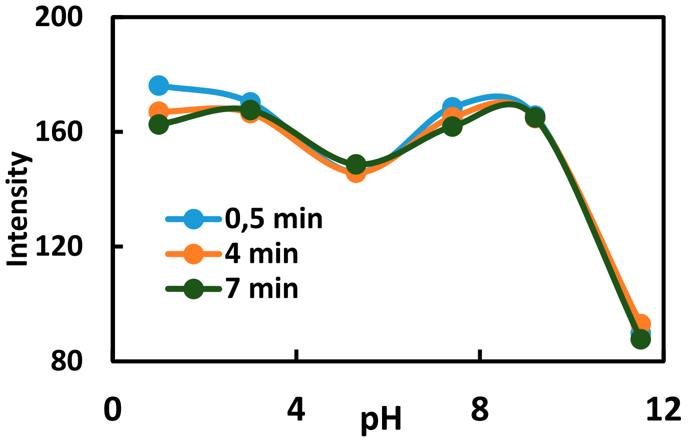

Figure S1. Red channel intensities for the visible photographs of the plate with *NaOCl – lysozyme – Cy5.5* mixture for the pH values from 1.0 to 11.5 and various reaction times. Other conditions as in Fig. 1.

a ***b***

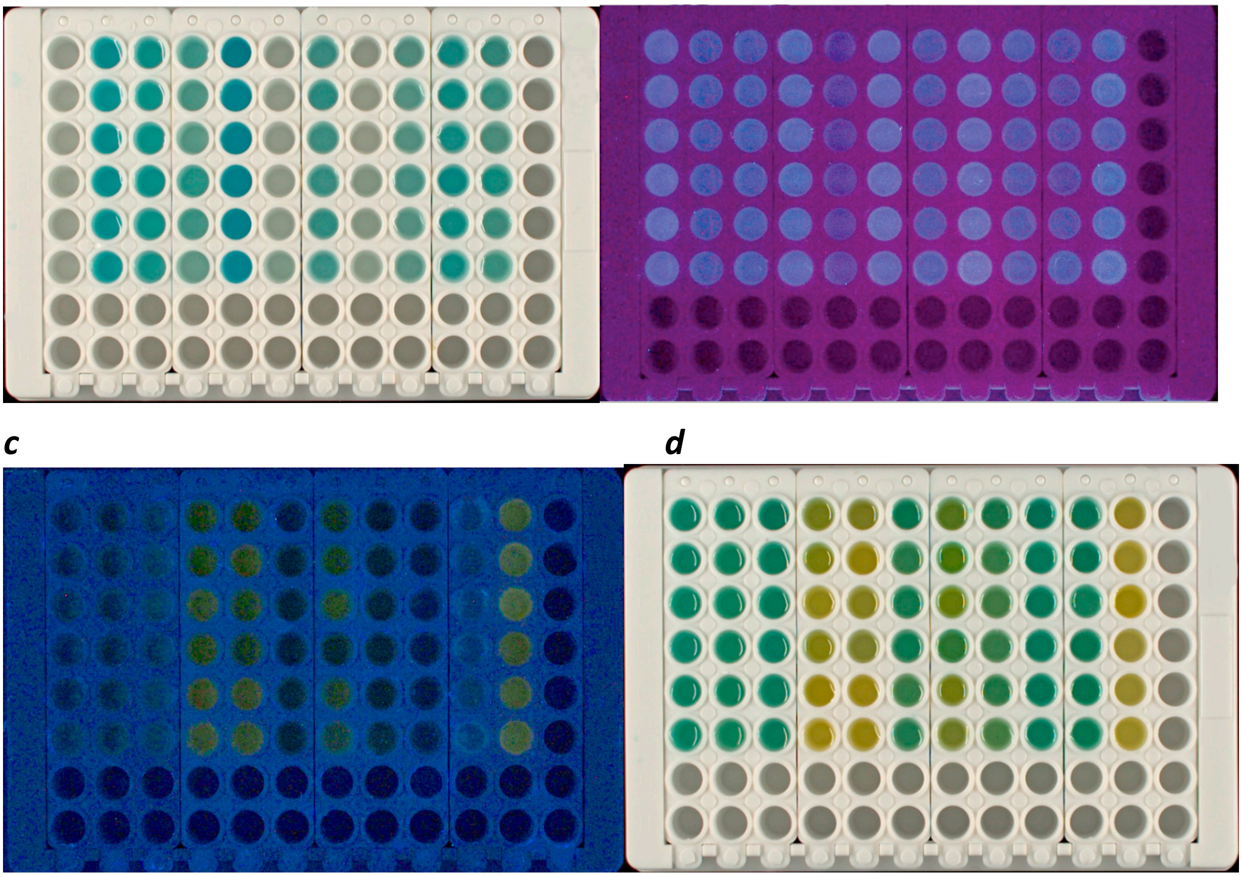

Figure S2. Images of the 96-well plates with the reaction mixtures of rennet samples with NaOCl and dyes: Cy5.5 (a, b) and IR-783 (c, d) in visible light (a, d) and under excitation with UV light at 254 nm (b) and 366 nm (c) captured 3 min (a, b) and 4 min (b, c) after the reaction start. Columns, from left to right: samples 1–10, blank.

Table S2. The amino acid composition of the proteins, mass %

Protein	Met	Cys	His	Trp	Tyr	Arg	Gly	Ref.
BSA	0.7	6.0	2.9	0.3	3.4	6.5	2.7	[1]
HSA	0.7	5.6	3.4	0.5	4.9	5.6	1.4	[2]
Lysozyme	1.6	6.2	0.8	4.7	2.3	8.5	9.3	[3]
γ-Globulin	0.9	2.1	2.4	2.6	5.8	4.0	3.4	[4]
Pepsin	1.5	4.7	0.8	4.7	11.6	1.6	11.1	[5]
Proteinase K	1.6	1.6	2.2	0.5	7.8	4.9	12.0	[6]
Chymosin	1.1	0.7	4.0	7.6	4.7	1.4	12.4	[7]

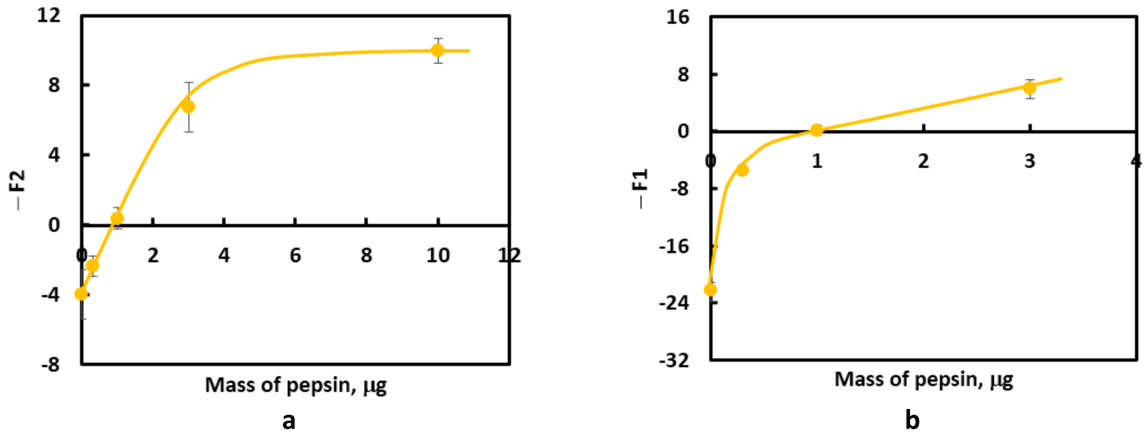

Figure S3. Dependences of LDA factors on the amount of pepsin per well: (a) on negative factor F2, (b) on negative factor F1.

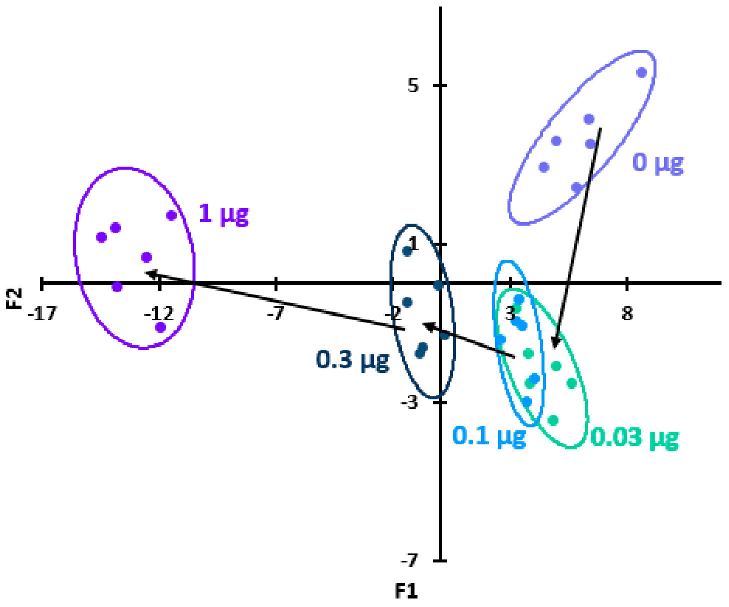

Figure S4. An LDA score plot for the low quantities of pepsin.

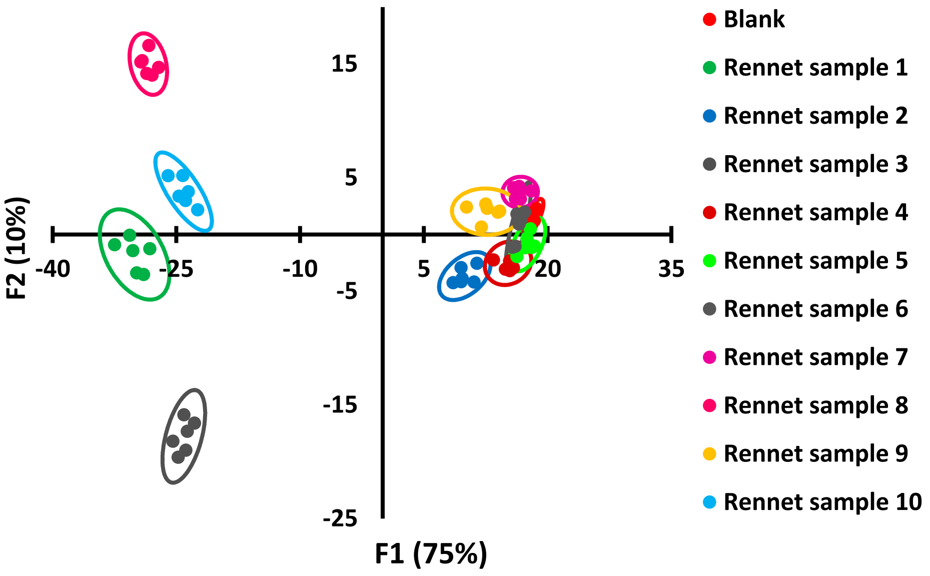

Figure S5. An LDA score plot for the discrimination of rennet samples, introduced as 1 g/L solutions (weight of sample/L).

Table S3. Full datasets used in this study. Each line represents a dataset obtained from a photograph (two right-hand columns), comments in the left-hand and central columns describe the dye and conditions of capturing the photos

I. Discrimination of model proteins

Dye	*Spectral conditions*	*Original dataset**	*Data columns left by the software after LDA procedure***
IR-783	Visible absorbance	1, R	
		1, G	
		1, B	1, B
		3, R	3, R
		3, G	
		3, B	3, B
		5, R	
		5, G	5, G
		5, B	
		9, R	9, R
		9, G	9, G
		9, B	
		21, R	
		21, G	
		21, B	
		51, R	
		51, G	51, G
		51, B	51, B
	Fluorescence excited by 366 nm light	1, R	
		1, G	
		1, B	
		3, R	3, R
		3, G	
		3, B	3, B
	Near-IR fluorescence	3	3
		6	6
Cy5.5	Visible absorbance	2, R	
		2, G	2, G
		2, B	2, B
		5, R	5, R
		5, G	
		5, B	
		8, R	
		8, G	8, G
		8, B	
		13, R	
		13, G	13, G
		13, B	

		27, R	27, R
		27, G	
		27, B	27, B
	Fluorescence excited by 254 nm light	2, R	2, R
		2, G	2, G
		2, B	2, B
	Fluorescence excited by 366 nm light	2, R	
		2, G	
		2, B	2, B
	Near-IR fluorescence	2	
		15	15

* Minutes from the reaction start and RGB channels are shown

**After sorting out insignificant data columns (variables)

II. Discrimination of rennet samples (1 g/L solutions)

Dye	*Spectral conditions*	*Original dataset*	*Data columns left by the software after LDA procedure*
IR-783	Visible absorbance	1, R	1, R
		1, G	1, G
		1, B	1, B
		2, R	2, R
		2, G	2, G
		2, B	2, B
		3, R	
		3, G	
		3, B	
		5, R	5, R
		5, G	
		5, B	5, B
		8, R	8, R
		8, G	8, G
		8, B	8, B
		18, R	18, R
		18, G	18, G
		18, B	
	Near-IR fluorescence	3	3
		7	
		20	20
Cy5.5	Visible absorbance	1, R	
		1, G	1, G
		1, B	1, B
		2, R	2, R
		2, G	
		2, B	2, B
		5, R	

		5, G	5, G
		5, B	5, B
	Fluorescence excited by 254 nm light	1, R	1, R
		1, G	1, G
		1, B	1, B
	Near-IR fluorescence	1	1

III. Discrimination of rennet samples with the 3 g/L solutions

Dye	*Spectral conditions*	*Original dataset*	*Data columns left by the software after LDA procedure*
IR-783	Visible absorbance	2, R	2, R
		2, G	
		2, B	2, B
		5, R	
		5, G	
		5, B	
		9, R	
		9, G	
		9, B	
		18, R	
		18, G	
		18, B	
		29, R	8, R
		29, G	29, G
		29, B	29, B
		40, R	40, R
		40, G	
		40, B	
	Fluorescence excited by 366 nm light	1	1
	Near-IR fluorescence	30	30
Cy5.5	Visible absorbance	1, R	1, R
		1, G	
		1, B	1, B
		7, R	7, R
		7, G	7, G
		7, B	
		18, R	18, R
		18, G	18, G
		18, B	18, B
	Fluorescence excited by 254 nm light	1, R	1, R
		1, G	1, G
		1, B	1, B
	Near-IR fluorescence	1	
		17	

Table S4. Accuracy of discrimination of rennet samples at 1 g/L for various datasets

Data set No	Data columns included in the original dataset						Number of data columns		Accuracy by cross-validation
	Cy5.5			IR-783					
	vis	UV (254)	NIR	vis	UV (366)	NIR	original	after DA	
1	+	+	+	+	+	+	34	23	92
2	+	-	+	+	-	+	31	24	91
3	+	-	-	+	-	-	27	23	91
4	+	-	+	+	-	+	27	23	91
5	-	-	-	+	-	+	21	13	85
6	-	-	-	+	-	-	18	14	87
7	+	-	+	-	-	-	10	9	75
8	-	-	+	-	-	+	4	3	69

References

1. Abdelmoez, W.; Yoshida, H. Production of Amino and Organic Acids from Protein Using Sub-Critical Water Technology. *Int. J. Chem. React. Engin.* **2013,** *11*, 1-16. DOI: 10.1515/ijcre-2013-0017.

2. Spahr, I. F.; Edsall, J.T. Amino acid composition of human and bovine serum mercaptalbumins. *J. Biol. Chem.* **1964**, *239*, 850 – 854. DOI: 10.1016/S0021-9258(18)51668-9.

3. Paice, M. G.; Jurasek, L. Structural and Mechanistic Comparisons of Some β-(1 → 4) Glycoside Hydrolases. Hydrolysis of Cellulose: Mechanisms of Enzymatic and Acid Catalysis. *Adv. Chem.* **1979,** *361* – 374. DOI: 10.1021/ba-1979-0181.ch018.

4. Crumpton, M.J.; Wilkinson, J.M. Amino acid compositions of human and rabbit gamma-globulins and of the fragments produced by reduction. *Biochem J.* **1963**, *88*, 228–234. DOI: 10.1042/bj0880228.

5. Mills, J.; Tang, J. Molecular weight and amino acid composition of human gastricsin and pepsin. *J. Biol. Chem.* **1967,** *242*, 3093–3097. DOI: 10.1016/S0021-9258(18)95937-5.

6. Dattagupta, J.K.; Fujiwara, T.; Grishin, E.V.; Lindner, K.; Manor, P.C.; Pieniazek, N.J.; Saenger, W.; Suck, D. Crystallization of the fungal enzyme proteinase K and amino acid composition. *J. Mol. Biol.* **1975,** *97*, 267–271. DOI: 10.1016/S0022-2836(75)80039-8

7. Yu, J.; Iwasaki, S.; Tamura, G.; Arima, K. Physical Properties and Amino Acid Composition of Mucor-rennin Crystal Isolated from Mucor pusillus var. Lindt. *Agric. Biol. Chem.* **1968**, *32*, 1051–1052. DOI: 10.1080/00021369.1968.10859184.
